# Barriers and Facilitators to Hepatitis C Virus Treatment in Los Angeles County

**DOI:** 10.21203/rs.3.rs-9025931/v1

**Published:** 2026-03-16

**Authors:** Shikha Handa, Nathan Sudeep, Larisa Albers, Monica Pattarroyo, Juan Vasquez, Roberto E. Montgomerie, Ryan M. Nguyen, Kendall Dimson, Deena Afana, Chrysovalantis Stafylis, Jeffrey D. Klausner, Daniel Soto

**Affiliations:** University of Southern California; University of Southern California; University of Southern California; University of Southern California; University of Southern California; University of Southern California; University of Southern California; University of Southern California; University of Southern California; University of Southern California; University of Southern California; University of Southern California

## Abstract

Diagnosed but untreated chronic Hepatitis C virus (HCV) infection is a major public health problem in Los Angeles (LA) County, particularly among underserved and minority groups. This mixed-methods study (October 2024-January 2025) used de-identified programmatic data from 344 case-residents in a linkage-to-cure program and conducted thematic analysis of semi-structured interviews with ten participants (seven untreated, three treated) from the linkage-to-cure program. By integrating these quantitative and qualitative findings, the study identified community-level and individual barriers and facilitators to HCV treatment. Quantitatively, 54% of case-residents reported barriers, such as personal barriers, provider-specific barriers, insurance barriers, and inadequate access to medical care. Qualitative themes expanded on these findings, identifying homelessness, incarceration, and drug addiction as key personal barriers. Provider-related barriers included language barriers, frustration in navigating the healthcare system, long wait times to see a provider, and participants’ lack of HCV information. Insurance barriers were characterized by cost barriers and inadequate access to medical care due to a lack of transportation. Facilitators of treatment included personal motivation to seek treatment, family and community-based support systems, trust in their medical provider, adequate insurance coverage, understanding the benefits of HCV treatment, and the desire to avoid stigmatization. Eliminating HCV as a public health problem requires equitable interventions that address individual and system-level barriers. These include implementing language support services, leveraging patient navigation and care coordination services, utilizing mobile clinics for the unhoused, ensuring treatment continuity for people re-entering the community from incarceration, and removing insurance restrictions, such as prior authorization requirements.

## Background

Chronic Hepatitis C virus (HCV) infection is an asymptomatic infectious disease that affects the liver. Globally, Hepatitis C is a major cause of liver transplants [1]. Hepatitis C is primarily a result of blood-to-blood transmission, spread by needles shared during intravenous drug usage [2]. Without treatment, HCV-infected individuals can develop liver failure, liver cancer, cirrhosis, and die prematurely [1]. HCV infection can be cured with eight to 12 weeks of daily oral treatment with the use of direct-acting antiviral medication.

### Hepatitis C Virus in Los Angeles County

More than five million Americans, or around two percent of the American adult population, have anti-HCV antibodies, suggesting prior or current infection [3]. In Los Angeles (LA) County, California, 27,678 people were diagnosed with HCV between 2019 and 2023, but only 30% of people reported knowledge of HCV treatment [4]. Treatment completion rates remain low, with only 32% of people completing HCV treatment [5]. Notably, non-White minority groups, particularly Hispanic, Latino, or Black, had the lowest percentages of reported treatment completion [5].

Recent policy changes in Medi-Cal, such as the removal of prior authorization and prescriber restrictions for HCV treatment [6], were intended to improve access. However, significant obstacles remain, including structural issues in the healthcare system, provider-related practices, and patient-level factors such as stigma [7]. In particular, gaps remain in understanding the unique experiences of Spanish-speaking Latinx individuals, who represent a significant proportion of the untreated population. Therefore, this study aimed to identify and describe the barriers and facilitators to HCV treatment among LA County residents.

## Methods

A mixed-methods study was conducted from October 2024 to January 2025.

### Participants and recruitment

In collaboration with the LA County Department of Public Health (DPH), the University of Southern California (USC) established a linkage-to-cure program that uses the LA County DPH’s HCV surveillance registry [8]. As part of the linkage-to-cure program, DPH case managers contact LA County residents who have a reported HCV-RNA-positive test result. Case managers provide HCV education and assess whether residents are aware of their diagnosis, have taken steps toward treatment, and are interested in linkage-to-cure services. Interested residents are linked to HCV treatment with their primary care provider, community health clinics or a telehealth program [8].

For the purposes of this study, we extracted and analyzed de-identified programmatic data to evaluate the obstacles of program participants during the analysis period. In addition, a subset of those contacted were invited to participate in a 30- to 60-minute semi-structured qualitative interview if (1) they had a confirmed HCV-RNA-positive result, (2) were LA County residents, and (3) were 18 years old or older. Ten people met the inclusion criteria and were interviewed. Relying on Saunders et al. [9], we felt ten participants were enough to reach thematic saturation. Interviews were conducted in English and Spanish.

### Data collection

For the quantitative analysis, anonymous data were extracted from the linkage-to-cure program between October 2024 and January 2025. Data extracted included demographics (sex, age group, race/ethnicity), health insurance type, treatment status, willingness to receive treatment, and specific reported HCV barriers to treatment.

Interview guides were developed using the Transtheoretical Model and Health Belief Model, which served as a starting point to help determine the reasons for wanting to test and manage HCV [10]. Interview guides were tailored to treated and untreated HCV study participants. Questions about treatment journey and medication adherence were asked to treated study participants, but not to untreated study participants. This allowed for the development of a targeted guide that assessed knowledge, attitudes, and beliefs among treated and untreated HCV study participants. Interview guides explored study participant-level treatment barriers, including stigma, insurance challenges, and health literacy, as well as facilitators such as strategies to improve treatment linkage. Interviews were conducted on Zoom by trained interviewers, audio-recorded, transcribed verbatim, and stored securely on the USC OneDrive. Data were deidentified to keep the study participants’ information confidential. Participants could use pseudonyms to protect their privacy and received a $100 electronic gift card upon completion.

### Data analysis

The quantitative analysis is presented with descriptive statistics, utilizing frequencies and percentages. Data were analyzed using SAS Software Version 9.4. For the qualitative part of the analysis, an inductive coding approach was employed to analyze interviews with untreated and treated study participants [11, 12]. Thematic analysis was conducted through iterative coding of interview transcripts from study participant groups using Dedoose Version 9.2.22, a cloud-based qualitative analysis platform. Codes were developed collaboratively through repeated immersion in the data, with themes refined through comparative analysis across participant narratives.

### Ethical Considerations

The study received USC Institutional Review Board (IRB) approval (UP-24–00296). All interview participants provided verbal informed consent before participation.

## Results

From October 2024 to January 2025, LA County DPH case managers contacted 344 people with a reported HCV-positive test result. Among them, 168 (49%) were already treated for Hepatitis C, and 176 had not received any treatment. Table 〿 indicates that 54% of the study participants faced barriers to Hepatitis C treatment [13]. The most reported barriers to treatment were personal barriers, namely that it was not a personal health priority to start treatment, followed by issues with the medical provider, such as incomplete follow-up after initial visit. Lack of insurance and inadequate access to medical care were also barriers to HCV treatment [13].

We completed individual interviews with untreated study participants (n = 7) and treated study participants (n = 3). Of the untreated study participants, three were Spanish speakers, and four were English speakers. All three treated study participants were English speakers.

### Hepatitis C Treatment Barriers Faced by Untreated English and Spanish-Speaking Study Participants

A total of twelve parent codes/themes were created for untreated study participant narratives (n = 7), and of those, barriers to linkage-to-cure include (1) the high cost of getting treated, (2) language barriers, (3) transportation to get to a medical provider, (4) lack of information, and (5) limited ability to navigate the healthcare system.

Cost barriers were most commonly cited, especially among the Spanish-speaking participants. Participants mentioned the high cost of insurance and personal or family financial instability that prevented them from initiating or continuing HCV treatment. Language barriers were also substantial concerns.

*“Since I don’t speak 100% English, I was treated at the hospital in Los Angeles and one doctor was a liver doctor, the other one was a hepatitis doctor, the other one was a cirrhosis doctor, and I didn’t understand very well what they were telling me. And the medications were in English, and so I withdrew a little bit and I didn’t want to take care of myself anymore because I didn’t understand anything. Now I just changed… to another primary care doctor who explains it well in Spanish and in 20 minutes, he explained the hepatitis C.”* (Untreated Spanish-speaking Study Participant #1)

Lack of information emerged as a significant theme across interviews, indicating that insufficient or unclear information about Hepatitis C and treatment options is a common experience. This theme reflects healthcare providers’ failure to adequately explain Hepatitis C, its implications, and available treatments. The prevalence of this theme highlights a critical gap in community-level and patient education and the absence of health communication that may be contributing to treatment avoidance or delay. This knowledge gap emerged as a significant barrier to treatment engagement among the interviewed participants.

*“I hadn’t heard so much about [HCV at the time of my diagnosis]… I don’t really know all that stuff about Hepatitis, what causes it or what is up with that.”* (Untreated Spanish-Speaking Study Participant #2)

*“Okay, all right, ‘cause I feel like a real dummy right now… I didn’t get this information [previously, but] even [the] little bit you’ve given me [during the interview], it makes sense.”* (Untreated English-speaking Study Participant #5)

Frustration with navigating the healthcare system emerged as another prominent theme, particularly among Spanish-speaking participants. This theme reflects feelings of dissatisfaction or annoyance regarding healthcare experiences. It also captures broader discontent with the quality of care provided.

*“I tried [to get treatment] before, but at least 3 or 4 times, and [they] all have been unsuccessful because of the blood draw process… I was an IV drug user, so I have collapsed veins… For test, [they need] sometimes two tests, not just one. I remember at one clinic, they were successful, albeit after a few tries, you know, of poking me with a needle. But the second [visit] was unsuccessful. And they needed that second blood test in order to prescribe medicine to treat it. And that second blood draw wasn’t successful.”* (Untreated English-speaking Study Participant #3)

Spanish-speaking untreated participants appear to experience heightened frustration with their healthcare experiences, suggesting potential language-related or cultural barriers in provider-patient relationships. This frustration may further discourage engagement with the healthcare system for Hepatitis C treatment. Participants described experiences where they felt dismissed, not taken seriously, or treated impersonally by healthcare providers. These negative interactions appeared to create lasting impressions that discouraged future treatment-seeking behavior.

### Facilitators of Hepatitis C Treatment Among Untreated English and Spanish-Speaking Study Participants

Hepatitis C treatment facilitators among untreated community members include (1) high motivation to seek treatment and (2) the importance of support systems.

The motivation to seek treatment captures the internal and external drivers encouraging patients to pursue care for their HCV infection. The motivation theme was particularly prominent among Spanish-speaking study participants.

*“Because, well yes, I have [HCV which] is going to make me sick in the future or I can pass [it] on to someone else. I think it is good to [seek treatment].”* (Untreated Spanish-speaking Study Participant #2)

Having a support system was important among participants. This theme encompasses the role of personal, family, and other social networks in influencing treatment decisions. Desire for provider support/guidance was most frequent, familial support and clinician trust were also commonly discussed, and religious support was the least mentioned. The distribution of support system codes showed interesting patterns across language groups. Familial support was more evenly distributed across both language groups, indicating that family influence on treatment decisions is important regardless of language or cultural background. Spanish-speaking participants more frequently mentioned the desire for provider support/guidance, suggesting that Spanish-speaking untreated individuals may particularly value and need additional guidance from healthcare providers.

*“I’ve been involved with the NA [Narcotics Anonymous] community. So that’s kind of like my support system, you know. We’re kind of open with each other, so there’s no judgment there, you know… they know my defects. We know each other’s, you know, defects and stuff. So that’s our support system. And I have some family, too, that understands what I’m going through.”* (Untreated English-speaking Study Participant #3)

### Hepatitis C Treatment Barriers Faced by Treated English-Speaking Study Participants

Among treated English-speaking study participants (n = 3), several treatment barriers emerged that prevented them from accessing timely HCV treatment. A total of six parent codes were created for treated study participant narratives, and of those, participants described four major barriers to HCV treatment that disrupted their treatment journey: (1) homelessness, (2) incarceration, (3) drug addiction, and (4) long wait times to see a provider.

For Treated English-speaking Study Participant #1, these challenges were deeply interconnected. He explained, *“Yeah, I bet I was homeless. I was in and out of jail… These are kind of all of the, you know, the consequences of drug addiction, you know, which is how I got the Hep C [through] sharing needles.”* His experience illustrates how overlapping life circumstances made it difficult to prioritize health or engage in care.

Treated English-speaking Study Participant #4 encountered structural barriers, such as delays in the referral process. *“I find Hepatitis C, and my primary doctor referred me for [a] specialist. It took so long time to meet me,”* she shared, expressing frustration that *“to start my medication, I took 2 months to meet the doctor.”* Her case underscores how even patients ready to begin treatment can face delays caused by fragmented care coordination and long waits for specialist access.

These barriers, whether rooted in social instability or healthcare system delays, significantly influenced access to timely and consistent Hepatitis C treatment.

### Facilitators of Hepatitis C Treatment Among Treated English-Speaking Study Participants

Despite their challenges, participants identified clear facilitators that helped them engage with care. These included (1) family support, (2) provider trust, (3) insurance coverage, (4) understanding the benefits of Hepatitis C treatment, and (5) the desire to avoid stigmatization.

Treated English-speaking Study Participant #1 emphasized how his wife helped keep him accountable. “She was always telling me, you know, *‘Did you take your meds?’… She wanted me to get healthy… because obviously she loves me, and she loves our family.”* Her encouragement made adherence feel like a shared responsibility rooted in love and concern. The birth of a child also served as a powerful motivator. The same treated participant described how fatherhood shifted his perspective: *“Once that happened, and then the birth of my son happened shortly after that. It just gave me this new lease on life, and was just like, hey man… you’re not getting younger. You need to get healthier. You need to be there. You need to be around for your kids.”*

He also reflected on the impact of provider communication. *“If they say this is a good idea, then I’m gonna do it,”* he said, underscoring how confidence in his care team helped him move forward with treatment.

Insurance played a critical role. Treated English-speaking Study Participant #1 shared, *“I’m just on MediCal, you know, regular medical insurance, and they covered it fully… everything was from the specialist to my GP… It was very easy… one of the easier things I had to do.”* For him, comprehensive Medi-Cal coverage eliminated financial stress and simplified care access.

After completing treatment, participants described the benefits of treatment, specifically the improvements to both their physical health and sense of self. Treated English-speaking Study Participant #4 said the medication allowed her to return to her normal routine. *“[I’ve] improved. I am back for the normal life,” she said. “I’m active now, I can do everything.”* She associated recovery with regaining control over daily life and being able to care for her family again. Treated English-speaking Study Participant #1 described a deep emotional shift. *“It made me feel like a real adult… I’m being responsible for my kids. I’m being responsible for my wife. I’m doing all these things, you know, after living a life of risk taking behavior that led to me getting a hep C.”* For him, treatment wasn’t just about eliminating the virus. It was about rebuilding a stable and purposeful life.

Participants also expressed that the stigma associated with Hepatitis C shaped their sense of urgency to pursue care. Treated English-speaking Study Participant #1 recalled avoiding physical contact, saying, *“I can’t shake your hand right now*… *Well, I’m bleeding*… *what does that mean?”* This discomfort reflected internalized judgment from others, even when the risk was low. The desire to eliminate ongoing stigmatization and embarrassment motivated them to seek treatment.

## Discussion

This mixed-methods study allowed for an in-depth exploration of barriers and facilitators to Hepatitis C treatment. A comparison of quantitative survey data and qualitative interview themes reveals critical areas of convergence and nuance regarding patient experience. This study highlights the complex interplay of linguistic, socioeconomic, and systemic factors that must be addressed to positively influence treatment engagement and outcomes.

### Linguistic Factors

Linguistic factors include barriers such as limited English proficiency. Quantitative responses indicated that personal and provider-specific barriers were the most significant barriers to care. Qualitative interviews expanded on this, identifying language barriers and frustration navigating the healthcare system as the specific drivers of negative provider interactions. While quantitative data show that people are highly motivated to seek treatment, qualitative themes suggest this motivation is often stifled by a lack of linguistic and cultural sensitivity. For instance, limited English proficiency is a barrier to Hepatitis C treatment among migrant populations. Spanish-speaking immigrants often report that a lack of translated materials and culturally sensitive services prevents them from understanding their risk or the benefits of newer treatments [14]. This is supported by a 2023 study of California safety-net clinics, which showed that nearly one in five Spanish-language calls ended with the scheduler hanging up or stating that no Spanish-speaking assistance was available [15]. Our findings that Spanish-speaking participants frequently experienced heightened frustration and a greater need for provider guidance aligned with prior evidence showing that personalized outreach in Spanish, such as for colorectal cancer [16], can significantly improve adherence and potentially bridge the Hepatitis C treatment gap.

### Socioeconomic Factors

Social support systems, financial instability, insurance status, housing security, and the impact of stigma significantly shape the landscape of Hepatitis C treatment engagement and continuation. This study’s findings emphasize the importance of social facilitators. Untreated study participants were highly motivated to seek treatment and cure their infection. This indicates an opportunity for healthcare providers to leverage motivation and convert it into treatment-seeking behavior. Outreach strategies should highlight the benefits of treatment to their health, i.e., the decreased likelihood of liver cancer, and the opportunity to protect loved ones from infection, which may convince people to get treatment. Evon et al. [17] identified several facilitators of treatment, including better future quality of life and achieving an HCV-uninfected status. Highlighting these comprehensive benefits in patient education and counseling could serve as powerful motivators for treatment initiation. Amoako et al. [18] reported that family, especially having children, shifts the perspective on patient health and acts as a facilitator of Hepatitis C treatment. This facilitation is driven by a sense of familial duty, where pursuing treatment is viewed as a way to fulfill one’s responsibilities to loved ones and ensure the well-being of the entire household [19]. Since family support played a crucial role in treatment decisions for treated study participants, outreach and education efforts should encourage the inclusion of family members in treatment discussions.

However, those social motivators can be undermined by the harsh realities of economic instability. Participants highlighted that being underinsured or uninsured, and overall financial instability, created significant challenges to initiating or continuing their care. These findings suggest that for many participants, the inability to navigate the costs associated with healthcare is a major deterrent to treatment engagement. Addressing these economic hurdles is essential for moving beyond diagnosis to treatment initiation.

The treatment journey is further complicated for those experiencing social instability, such as homelessness or incarceration. For participants facing those challenges, often in tandem with drug addiction, Hepatitis C care is frequently sidelined by the more immediate demands of basic survival and the consequences of the legal system. Furthermore, qualitative insights revealed that while stigma can act as a motivator for some seeking to eliminate a source of judgment [18], it remains a deterrent among immigrant populations [20]. To address those barriers, providers should work closely with patient navigators, strengthen harm reduction practices, utilize mobile clinics for the unhoused, ensure treatment continuity for people re-entering the community from incarceration, and policies should reflect up-to-date staff training on building trust and cultural sensitivity when working with people with Hepatitis C.

### Systemic Factors

Systemic factors included structural barriers within the healthcare system, such as insurance restrictions and treatment prior authorization requirements, and challenges in navigating complex care pathways. Insurance barriers were identified as obstacles in both the survey and interview data. Even with insurance (private insurance, Medicare, Medicaid), treatment initiation was low. Research shows that those with Medicaid face longer waiting times for care than those with private coverage, suggesting that insurance type and status are critical determinants of timely Hepatitis C treatment [21]. This is further evidenced by findings from LA County, where 69% of Hepatitis C-infected residents remained untreated, with the highest lack of treatment observed among those with no insurance or public insurance. While California removed prior authorization for California’s Medicaid program in 2024, Medicaid beneficiaries remain disproportionately disadvantaged due to high rates of co-occurring challenges like housing instability and substance use disorders. Consequently, Hepatitis C treatment initiation remains a significant challenge unless prior authorization is eliminated from all insurance types and underlying social determinants of health are addressed [22].

### Implications for Clinical Practice and Policy

Our findings have important implications for clinical practice and public health policy in LA County. Healthcare systems must prioritize patient navigation and care coordination services, comprehensive language support services, including professional medical interpretation, and culturally tailored patient education materials. Culturally competent healthcare staff is also important. Addressing negative healthcare experiences through improved provider communication and patient-centered care approaches could enhance treatment engagement and reduce healthcare system avoidance. Policy interventions focused on expanding insurance coverage, reducing treatment costs, and eliminating prior authorization requirements could significantly improve access to Hepatitis C treatment. By understanding specific local barriers, we can more effectively inform and strengthen local Hepatitis C elimination efforts, such as the LA County Viral Hepatitis Action Plan [23].

### Limitations

The small sample size may limit the generalizability of our findings to the broader population of LA County. Future research should include larger, more diverse samples to better understand the full spectrum of barriers and facilitators affecting Hepatitis C treatment engagement. While the inclusion of Spanish-speaking participants represents a significant strength of this study - reflecting a critical demographic within LA County - this focus may not fully capture the experiences of other linguistic minorities in the region, necessitating future research into diverse immigrant and refugee communities to develop a more comprehensive understanding of the linguistic and cultural factors affecting HCV treatment.

## Figures and Tables

**Table 〿.** Demographic Characteristics of Hepatitis C Linkage-to-Cure Contacts (n=344) with HCV RNA-Positive Test Result, Los Angeles County, October 2024 to January 2025

Characteristics	Residents (n=344)

**Sex**	
Male	232 (67%)

**Age**	
18–29 years old	7 (2%)
30–44 years old	60 (17%)
45–64 years old	147 (43%)
65+ years old	130 (38%)

**Race/Ethnicity**	
Asian	10 (3%)
Hispanic/Latino	98 (28%)
Black	64 (19%)
White	95 (28%)
Native American/Pacific Islander	1 (0%)
Multirace	1 (0%)
Other or Unknown	74 (22%)

**Health Insurance**	
Private Insurance	72 (21%)
Medicare	32 (9%)
Medicaid/Medi-Cal	92 (27%)
Other Public-sponsored insurance	17 (5%)
None or Unknown Insurance	24 (7%)
Decline to Answer	107 (31%)

**Treatment Status**	
Treated	168 (49%)

**Interest in receiving treatment**	214 (62%)

**Did you experience any barriers accessing/attempting to access treatment for Hepatitis C (n=197)**	

Yes	106 (54%)
No	65 (33%)
Decline to answer	26 (13%)

**Specific Barriers to Treatment (n=91)**	

**Personal**	39 (43%)
Not feeling any symptoms of infection	9
Not a personal health priority to start treatment	12
Concerned about treatment side effects	4
Incarceration	3
Other health priorities	6
Incomplete follow-up	3
Unaware of treatment options	2

**Insurance (**Underinsured/uninsured for treatment)	12 (13%)

**Provider-Specific**	28 (31%)
Provider did not recommend treatment	12
Incomplete follow-up after initial visit	15
Wrong lab results	1

**Access to Medical Care**	12 (13%)
I don’t have access to a doctor or a specialist who can treat Hepatitis C infection	7
Lack of transportation	2
Other	3

## Data Availability

The quantitative table generated during the study is available in the Figshare repository. This can be accessed via DOI: 10.6084/m9.figshare.31476604. For peer review purposes, the data can be accessed privately at: https://figshare.com/s/59c89223502c841d7f42.
